# Supplementary material for: Dietary Oat Bran Reduces Systemic Inflammation in Mice Subjected to Pelvic Irradiation
Source: Nutrients. 2020 Jul 22;12(8):2172. doi: 10.3390/nu12082172 (PMC7468988; doi:10.3390/nu12082172)
Supplement: Supplementary file 1 [file nutrients-12-02172-s001.pdf]

Supplementary Tables

Table S1. Composition of the High-oat and No-fiber diets.

Ingredient (%)	High-oat (15% fiber)	No-fiber (0% fiber)
Bioprocessed oat bran	28.8	0
Corn starch	4.7	33.5
Basal mixture	66.5	66.5
**Total**	**100**	**100**

Table S2. Composition of basal mixture.

Basal mixture ingredient (g/kg of total diet in dwb*)	High-oat	No-fiber
Casein	133	133
DL-methionine	2	2
Corn starch	250	250
Maltodextrin	87	87
Sucrose	106	106
Olive oil	47	47
Vitamin mixture	10	10
Choline bitartrate	2	2
TBHQ[#]	0.01	0.01
Mineral mixture	13	13
Calcium phosphate	11	11
Calcium carbonate	4	4

*Dry-weight basis. [#]Tertiary butylhydroquinone

Supplementary Figures

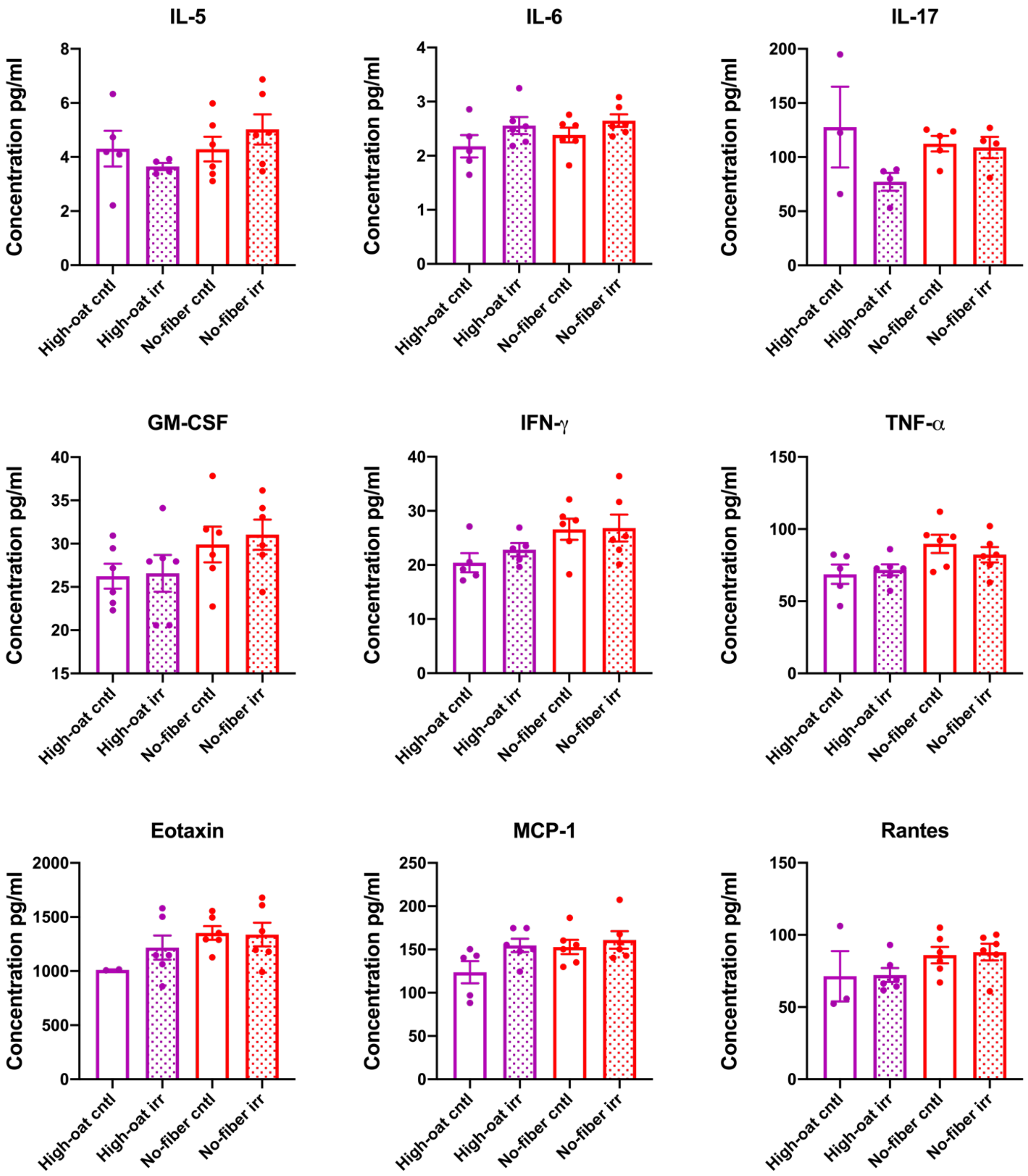

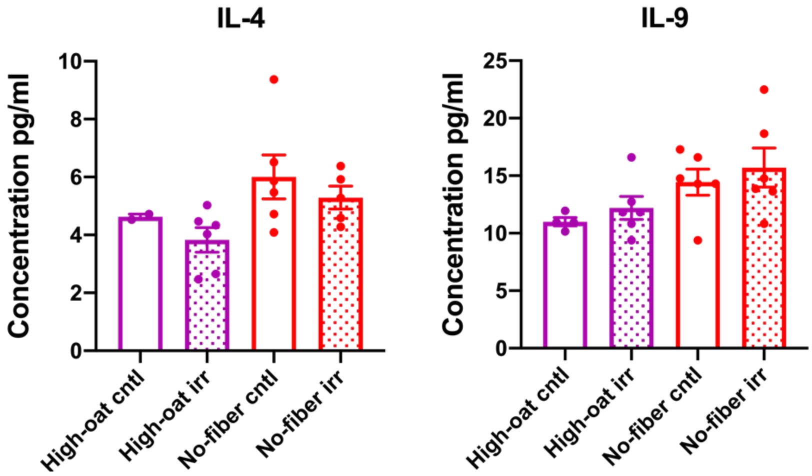

Figure S1. Serum cytokine and chemokine levels in mice at 1 week post-irradiation. A two-tailed Mann–Whitney test was used to compare the cytokine and chemokine levels in High-oat irr vs. High-oat cntl, No-fiber irr vs. No-fiber cntl, High-oat irr vs. No-fiber irr, and High-oat cntl vs. No-fiber cntl groups. Data shown are average concentrations and the error bars represent SEM.

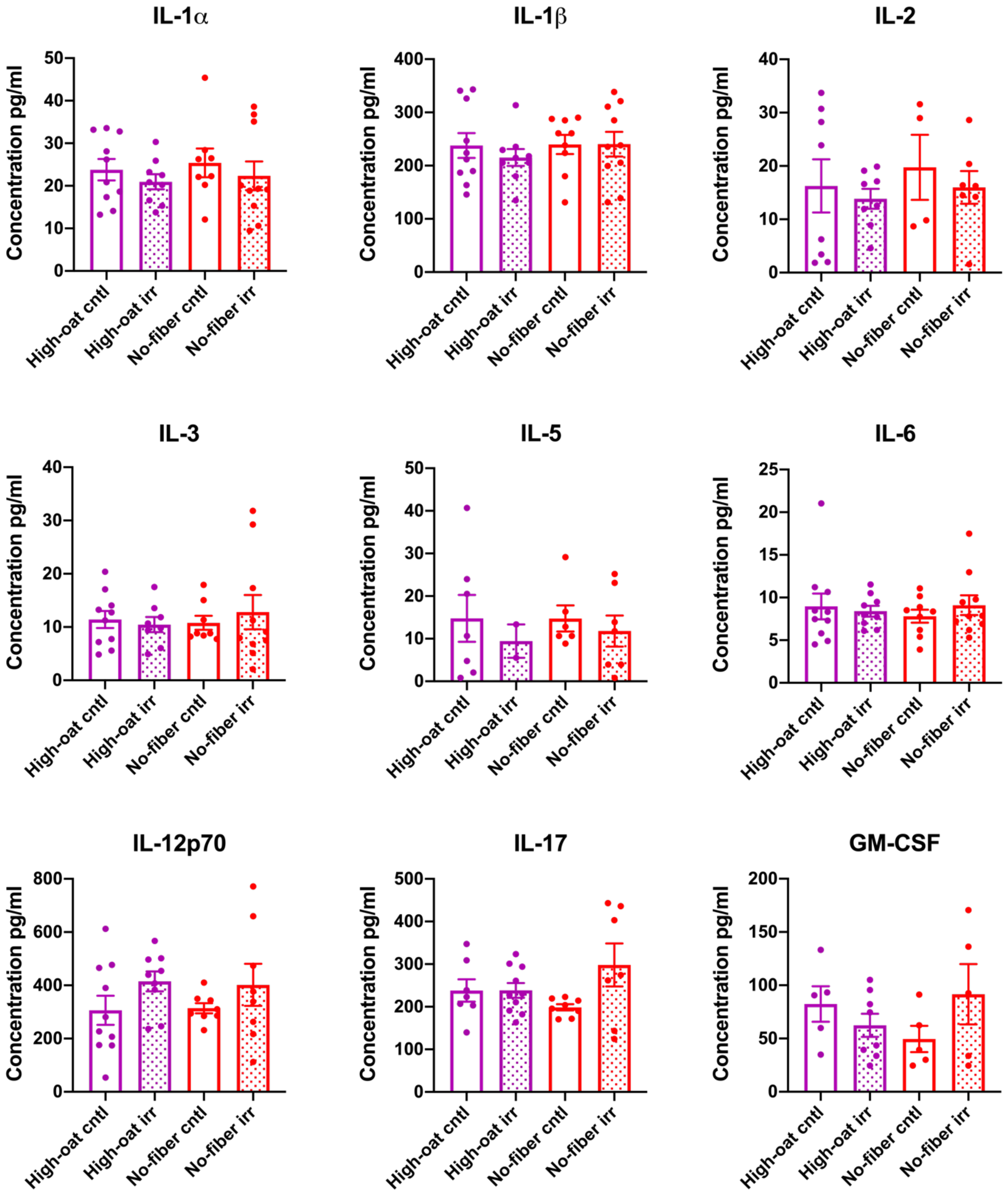
IL-1α
Concentration pg/ml
0
10
20
30
40
50
High-oat cntl
High-oat irr
No-fiber cntl
No-fiber irr
IL-1β
Concentration pg/ml
0
100
200
300
400
High-oat cntl
High-oat irr
No-fiber cntl
No-fiber irr
IL-2
Concentration pg/ml
0
10
20
30
40
High-oat cntl
High-oat irr
No-fiber cntl
No-fiber irr
IL-3
Concentration pg/ml
0
10
20
30
40
High-oat cntl
High-oat irr
No-fiber cntl
No-fiber irr
IL-5
Concentration pg/ml
0
10
20
30
40
50
High-oat cntl
High-oat irr
No-fiber cntl
No-fiber irr
IL-6
Concentration pg/ml
0
5
10
15
20
25
High-oat cntl
High-oat irr
No-fiber cntl
No-fiber irr
IL-12p70
Concentration pg/ml
0
200
400
600
800
High-oat cntl
High-oat irr
No-fiber cntl
No-fiber irr
IL-17
Concentration pg/ml
0
100
200
300
400
500
High-oat cntl
High-oat irr
No-fiber cntl
No-fiber irr
GM-CSF
Concentration pg/ml
0
50
100
150
200
High-oat cntl
High-oat irr
No-fiber cntl
No-fiber irr

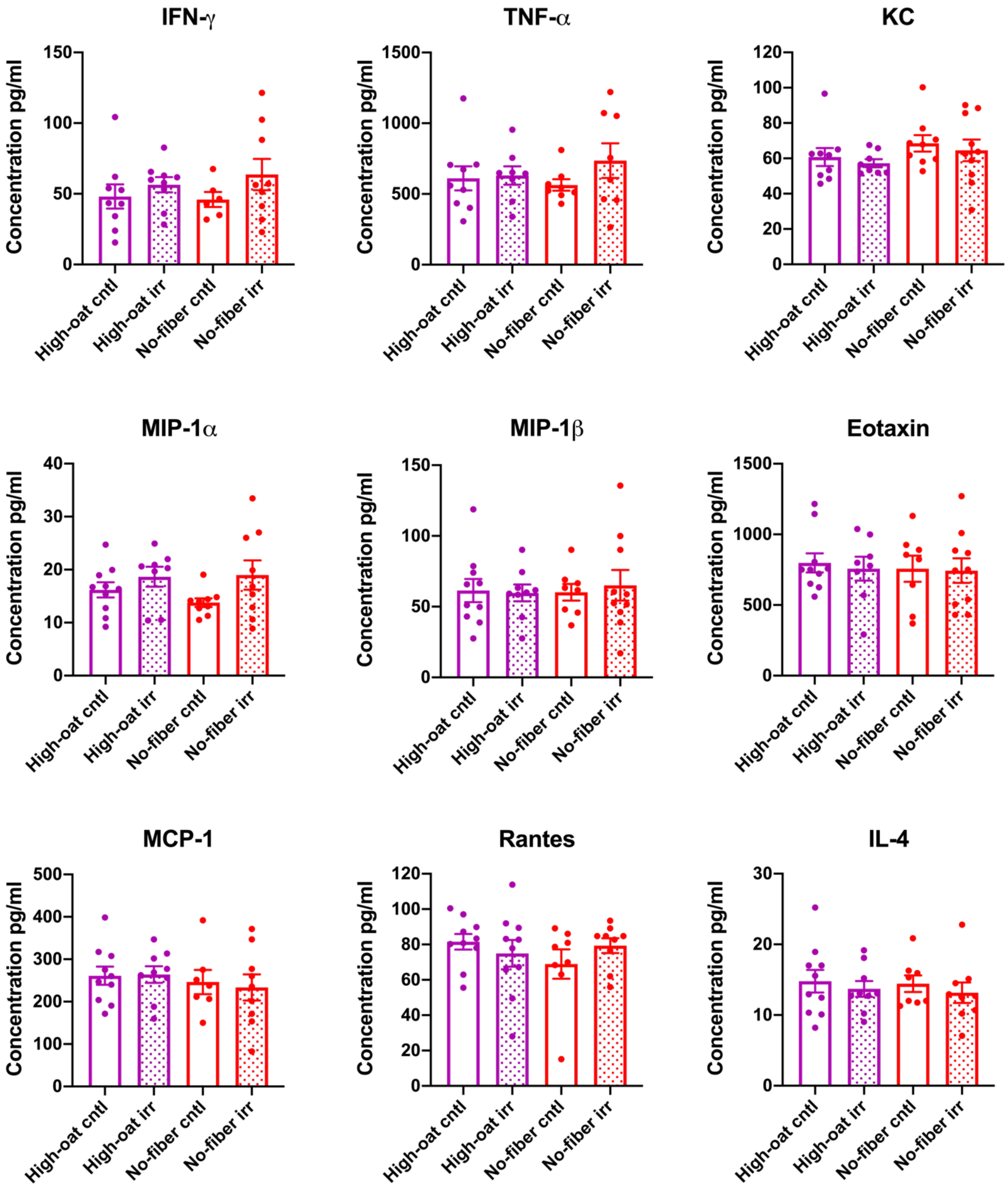
IFN-γ
TNF-α
KC
MIP-1α
MIP-1β
Eotaxin
MCP-1
Rantes
IL-4
Concentration pg/ml
High-oat cntl
High-oat irr
No-fiber cntl
No-fiber irr

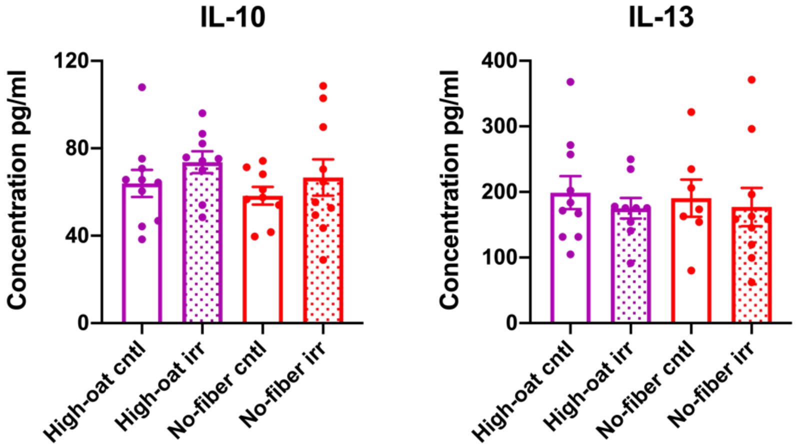

Figure S2. Serum cytokine and chemokine levels in mice at 6 weeks post-irradiation. A two-tailed Student's *t*-test was used to compare the levels of IL-1α, IL-1β, IL-3, IL-10, IL-12p70, eotaxin, MIP-1α, and MIP-1β in the High-oat irr vs. High-oat cntl, No-fiber irr vs. No-fiber cntl, High-oat irr vs. No-fiber irr, and High-oat cntl vs. No-fiber cntl groups. A Mann–Whitney test was used to compare the levels of IL-2, IL-4, IL-5, IL-6, IL-13, IL-17, GM-CSF, IFN-γ, KC, MCP-1, RANTES, and TNF-α in the High-oat irr vs. High-oat cntl, No-fiber irr vs. No-fiber cntl, High-oat irr vs. No-fiber irr, and High-oat cntl vs. No-fiber cntl groups. Data are shown as averages and the error bars represent SEM.

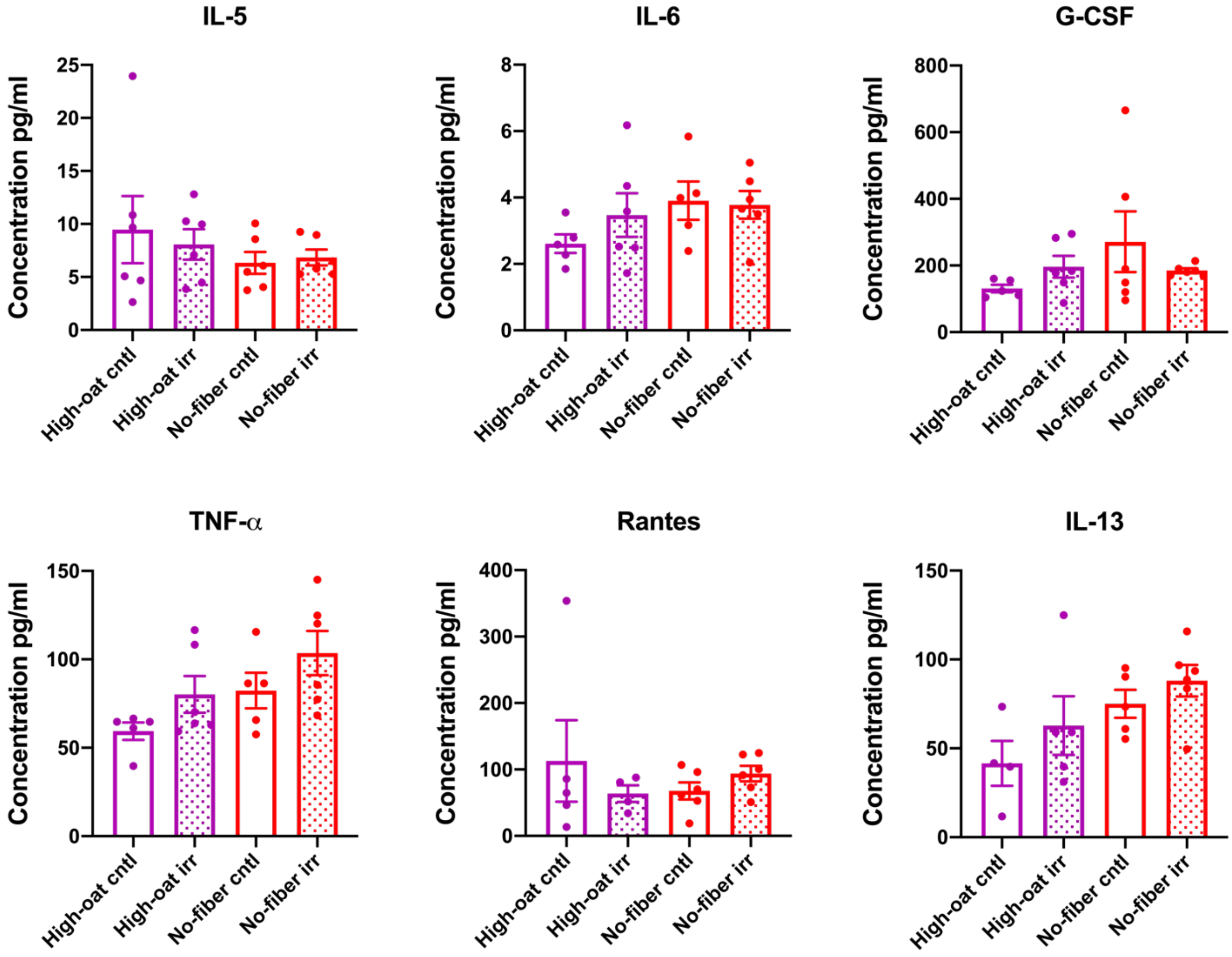

Figure S3. Serum cytokine and chemokine levels in mice at 18 weeks post-irradiation. A two-tailed Mann–Whitney test was used to compare the cytokine and chemokine levels in High-oat irr vs. High-oat cntl, No-fiber irr vs. No-fiber cntl, High-oat irr vs. No-fiber irr, and High-oat cntl vs. No-fiber cntl groups. Data shown are average concentrations and the error bars represent SEM.

A

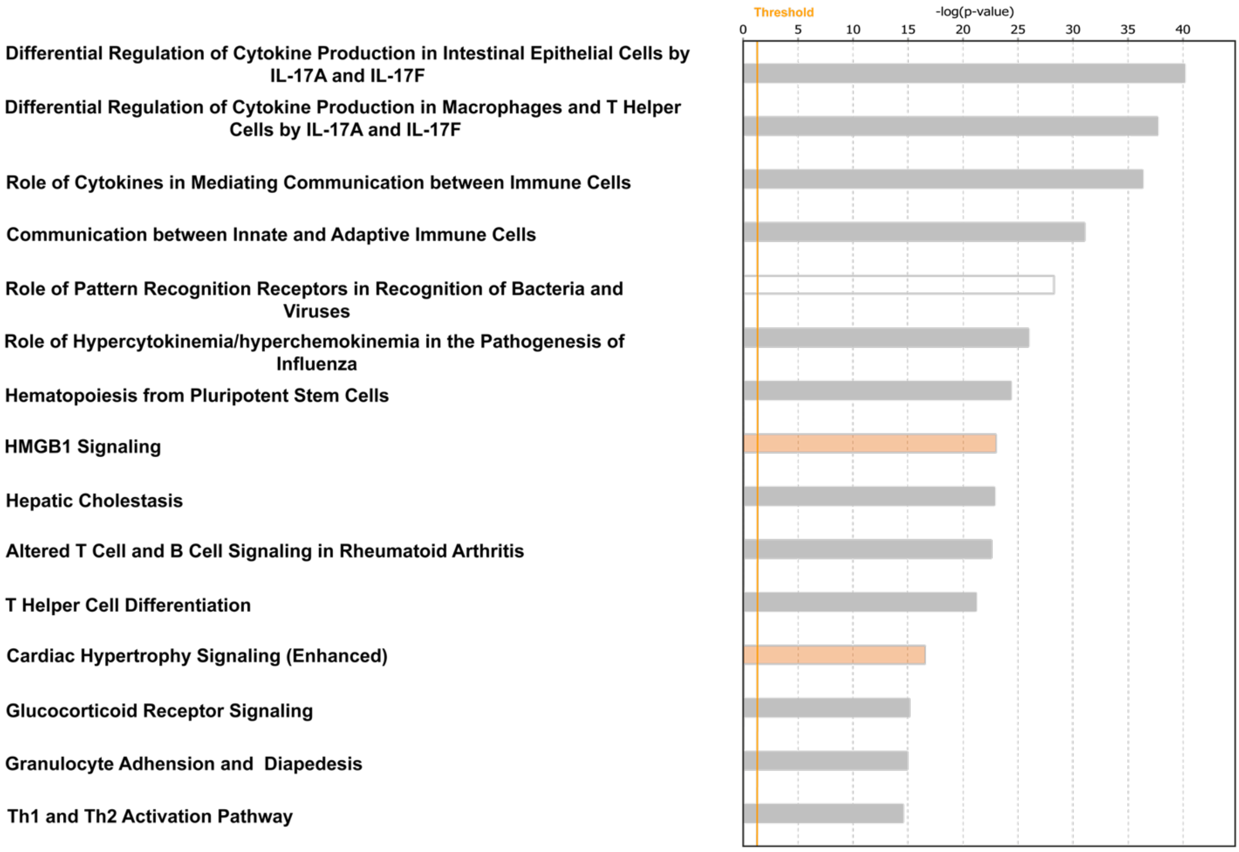
Threshold
-log(p-value)
0
5
10
15
20
25
30
35
40
Differential Regulation of Cytokine Production in Intestinal Epithelial Cells by IL-17A and IL-17F
Differential Regulation of Cytokine Production in Macrophages and T Helper Cells by IL-17A and IL-17F
Role of Cytokines in Mediating Communication between Immune Cells
Communication between Innate and Adaptive Immune Cells
Role of Pattern Recognition Receptors in Recognition of Bacteria and Viruses
Role of Hypercytokinemia/hyperchemokinemia in the Pathogenesis of Influenza
Hematopoiesis from Pluripotent Stem Cells
HMGB1 Signaling
Hepatic Cholestasis
Altered T Cell and B Cell Signaling in Rheumatoid Arthritis
T Helper Cell Differentiation
Cardiac Hypertrophy Signaling (Enhanced)
Glucocorticoid Receptor Signaling
Granulocyte Adhension and Diapedesis
Th1 and Th2 Activation Pathway

B

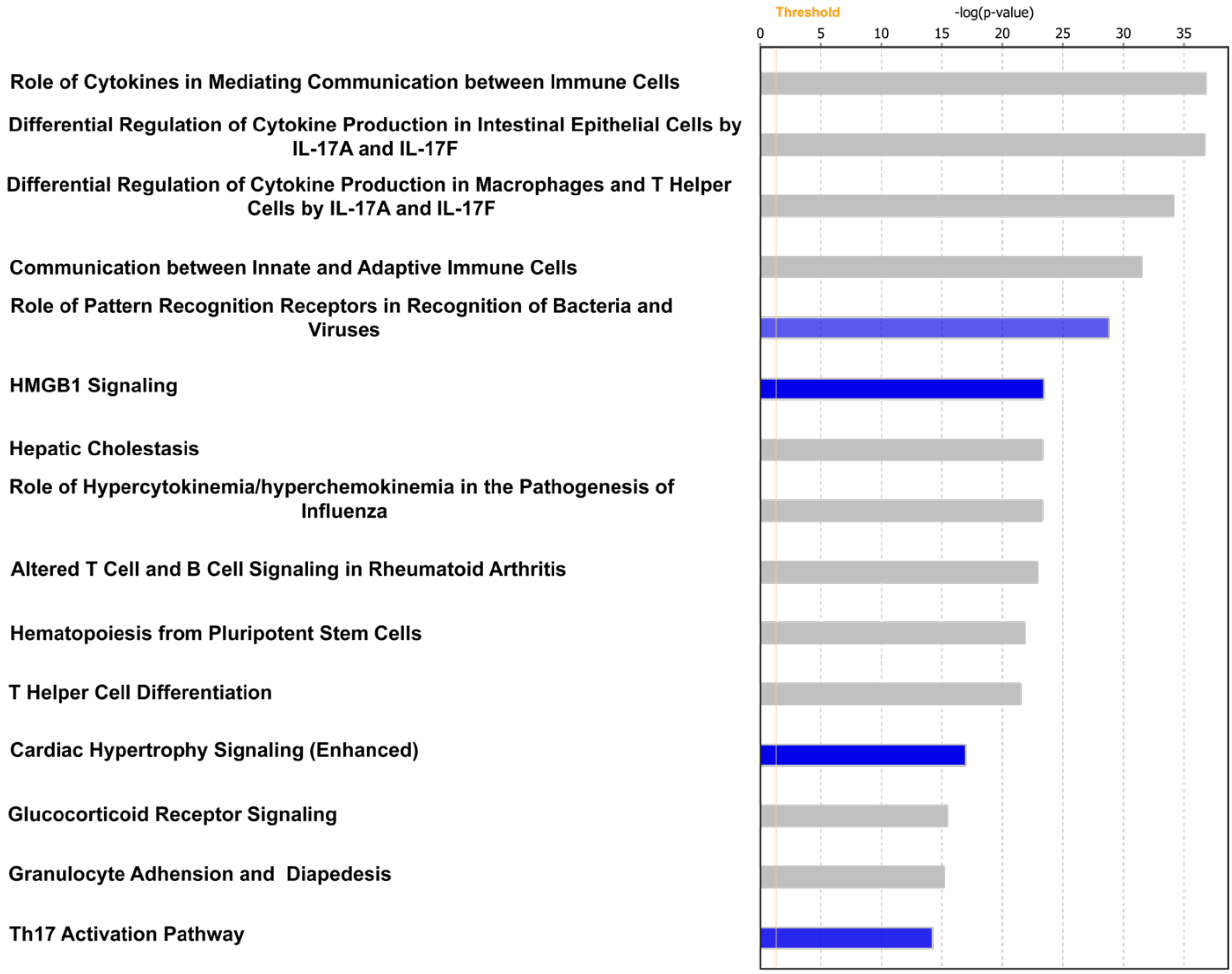

Threshold
-log(p-value)
0
5
10
15
20
25
30
35
Role of Cytokines in Mediating Communication between Immune Cells
Differential Regulation of Cytokine Production in Intestinal Epithelial Cells by IL-17A and IL-17F
Differential Regulation of Cytokine Production in Macrophages and T Helper Cells by IL-17A and IL-17F
Communication between Innate and Adaptive Immune Cells
Role of Pattern Recognition Receptors in Recognition of Bacteria and Viruses
HMGB1 Signaling
Hepatic Cholestasis
Role of Hypercytokinemia/hyperchemokinemia in the Pathogenesis of Influenza
Altered T Cell and B Cell Signaling in Rheumatoid Arthritis
Hematopoiesis from Pluripotent Stem Cells
T Helper Cell Differentiation
Cardiac Hypertrophy Signaling (Enhanced)
Glucocorticoid Receptor Signaling
Granulocyte Adhension and Diapedesis
Th17 Activation Pathway

C

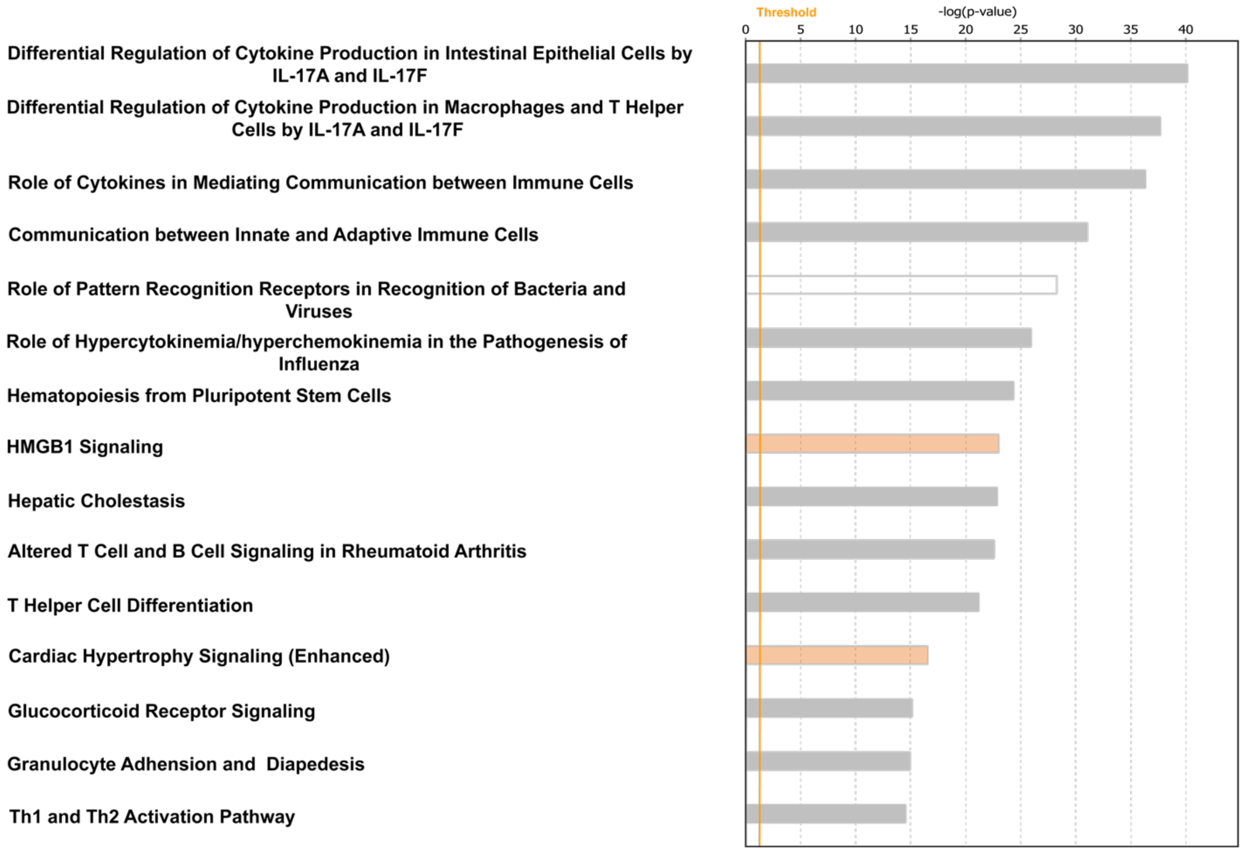

Figure S4. Pathway enrichment analysis. The top 15 canonical pathways associated with the cytokine profiles at (**A**) 1, (**B**) 6, and (**C**) 18 weeks post-irradiation, as determined by IPA. The bars indicate -log(*p*-values) calculated using Fisher's exact test, with the threshold set at $p = 0.05$.

A

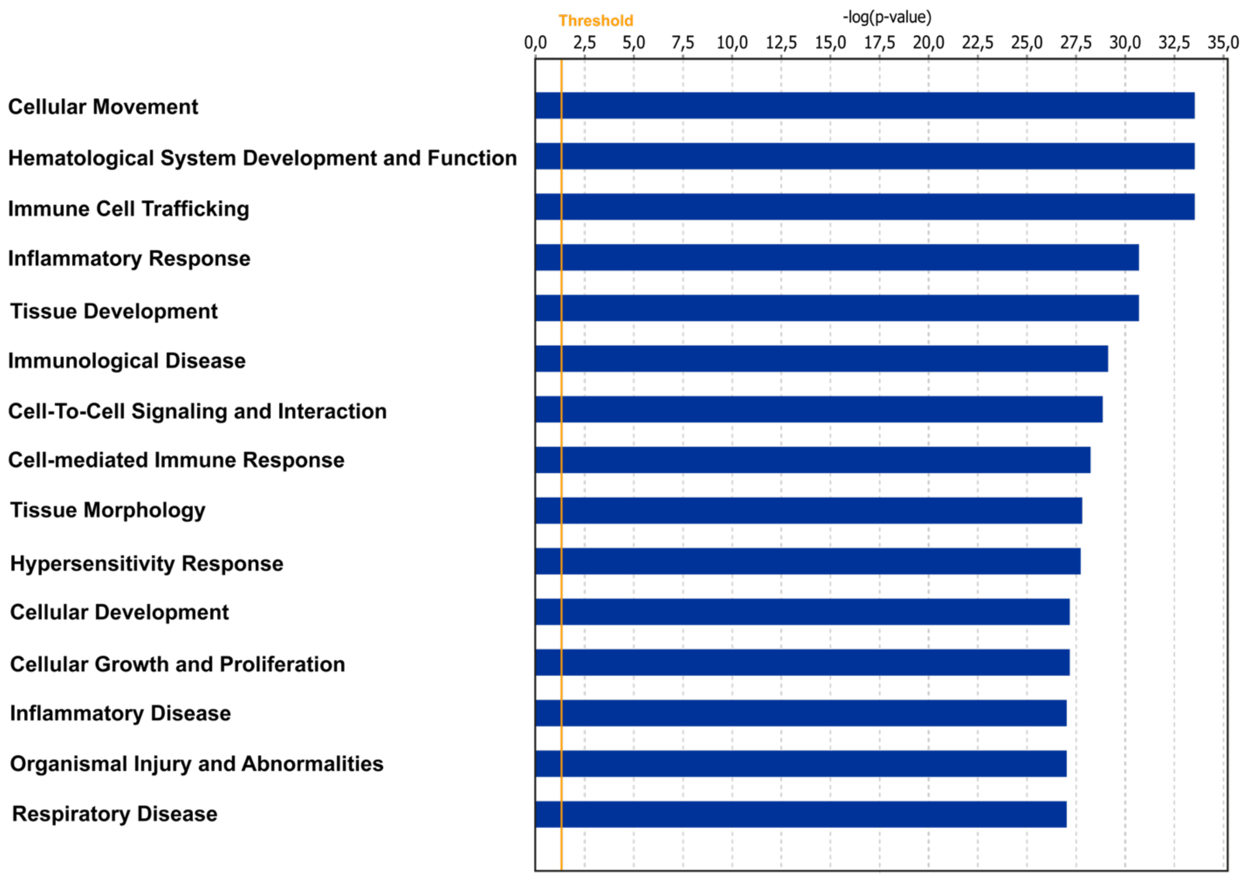
Threshold
-log(p-value)
0,0 2,5 5,0 7,5 10,0 12,5 15,0 17,5 20,0 22,5 25,0 27,5 30,0 32,5 35,0
Cellular Movement
Hematological System Development and Function
Immune Cell Trafficking
Inflammatory Response
Tissue Development
Immunological Disease
Cell-To-Cell Signaling and Interaction
Cell-mediated Immune Response
Tissue Morphology
Hypersensitivity Response
Cellular Development
Cellular Growth and Proliferation
Inflammatory Disease
Organismal Injury and Abnormalities
Respiratory Disease

B

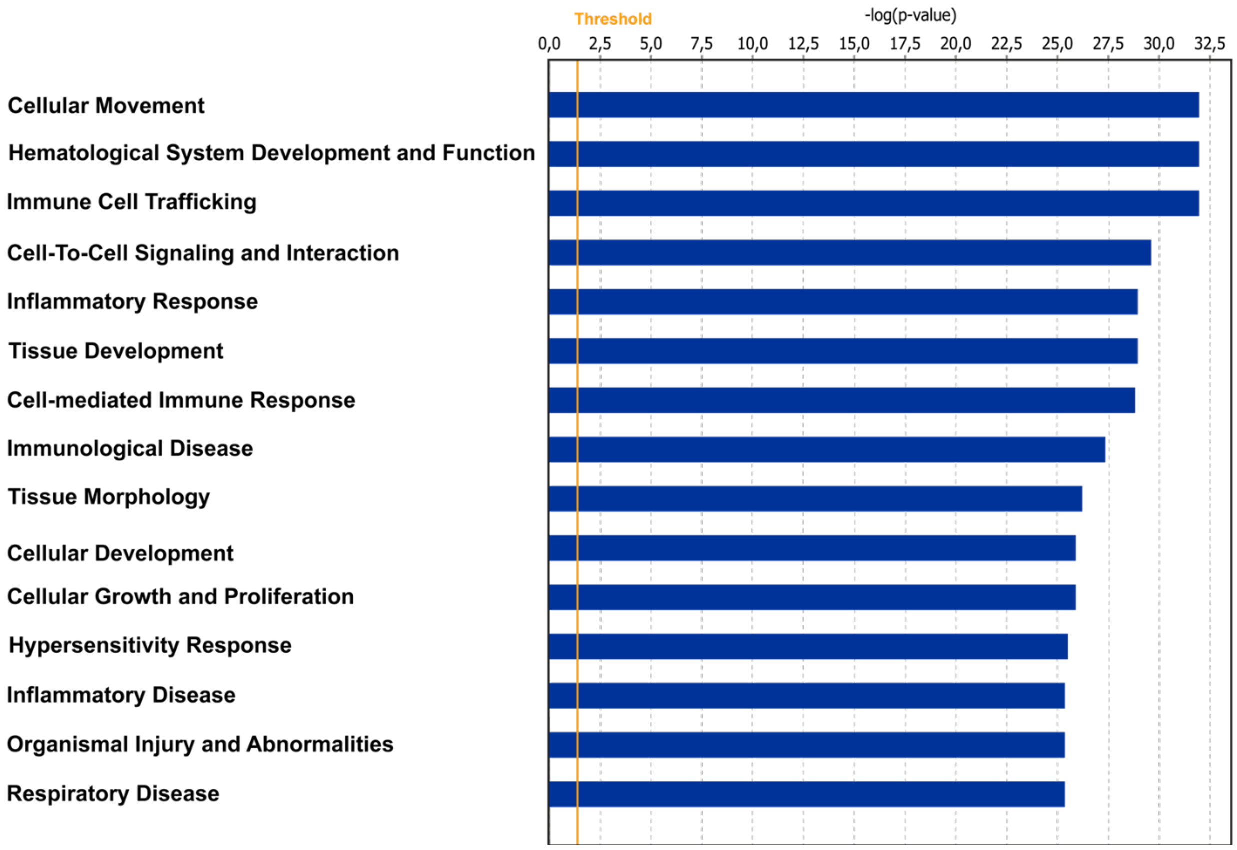
Threshold
-log(p-value)
0,0 2,5 5,0 7,5 10,0 12,5 15,0 17,5 20,0 22,5 25,0 27,5 30,0 32,5
Cellular Movement
Hematological System Development and Function
Immune Cell Trafficking
Cell-To-Cell Signaling and Interaction
Inflammatory Response
Tissue Development
Cell-mediated Immune Response
Immunological Disease
Tissue Morphology
Cellular Development
Cellular Growth and Proliferation
Hypersensitivity Response
Inflammatory Disease
Organismal Injury and Abnormalities
Respiratory Disease

C

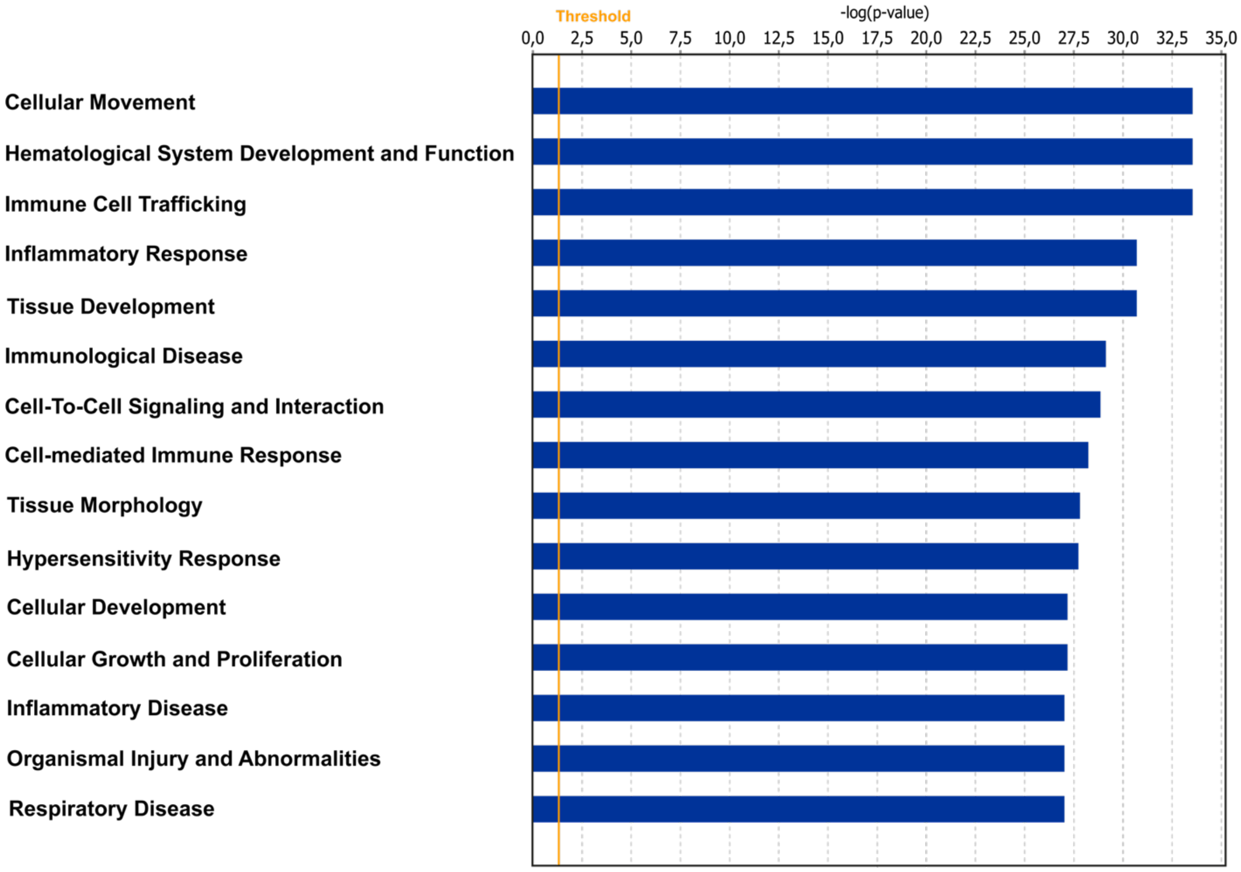

Figure S5. Biological function and disease enrichment analysis. The top 15 biological functions or diseases associated with the cytokine profiles at (**A**) 1, (**B**) 6, and (**C**) 18 weeks post-irradiation, as determined by IPA. The bars indicate -log(p-values) calculated using Fisher's exact test, with the threshold set at $p = 0.05$.
